# Tumor slices as a model to evaluate doxorubicin *in vitro *treatment and expression of trios of genes PRSS11, MTSS1, CLPTM1 and PRSS11, MTSS1, SMYD2 in canine mammary gland cancer

**DOI:** 10.1186/1751-0147-50-27

**Published:** 2008-07-04

**Authors:** Renata A Sobral, Suzana T Honda, Maria Lucia H Katayama, Helena Brentani, M Mitzi Brentani, Diogo FC Patrão, Maria Aparecida AK Folgueira

**Affiliations:** 1 Departamento de Radiologia e Cancerologia, Faculdade de Medicina da Universidade de São Paulo, São Paulo, Brasil; 2 Departamento de Bioinformática, Hospital do Câncer A.C. Camargo, São Paulo, Brasil

## Abstract

**Background:**

In women with breast cancer submitted to neoadjuvant chemotherapy based in doxorubicin, tumor expression of groups of three genes (PRSS11, MTSS1, CLPTM1 and PRSS11, MTSS1, SMYD2) have classified them as responsive or resistant. We have investigated whether expression of these trios of genes could predict mammary carcinoma response in dogs and whether tumor slices, which maintain epithelial-mesenchymal interactions, could be used to evaluate drug response *in vitro*.

**Methods:**

Tumors from 38 dogs were sliced and cultured with or without doxorubicin 1 μM for 24 h. Tumor cells were counted by two observers to establish a percentage variation in cell number, between slices. Based on these results, a reduction in cell number between treated and control samples ≥ 21.7%, arbitrarily classified samples, as drug responsive. Tumor expression of PRSS11, MTSS1, CLPTM1 and SMYD2, was evaluated by real time PCR. Relative expression results were then transformed to their natural logarithm values, which were spatially disposed according to the expression of trios of genes, comprising PRSS11, MTSS1, CLPTM1 and PRSS11, MTSS1, SMYD2. Fisher linear discrimination test was used to generate a separation plane between responsive and non-responsive tumors.

**Results:**

Culture of tumor slices for 24 h was feasible. Nine samples were considered responsive and 29 non-responsive to doxorubicin, considering the pre-established cut-off value of cell number reduction ≥ 21.7%, between doxorubicin treated and control samples. Relative gene expression was evaluated and tumor samples were then spatially distributed according to the expression of the trios of genes: PRSS11, MTSS1, CLPTM1 and PRSS11, MTSS1, SMYD2. A separation plane was generated. However, no clear separation between responsive and non-responsive samples could be observed.

**Conclusion:**

Three-dimensional distribution of samples according to the expression of the trios of genes PRSS11, MTSS1, CLPTM1 and PRSS11, MTSS1, SMYD2 could not predict doxorubicin *in vitro *responsiveness. Short term culture of mammary gland cancer slices may be an interesting model to evaluate chemotherapy activity.

## Introduction

Human and canine malignant mammary tumors share some epidemiological and clinicopathological features. Incidence in both species increases with age, lifetime exposure to endogenous or exogenous estrogens is a common risk factor, and the majority of malignant mammary gland tumors arises from epithelial tissue [1-3]. In addition, some prognostic factors are similar for both species, such as clinical stage, tumor size, histological type and grade, however adjacent lymph node involvement is still a matter of discussion [1,4-7]. Furthermore, estrogen receptor expression, proliferation index evaluated by PCNA, Ki67 expression, or S-phase rate, have also been correlated to prognosis in canine mammary tumors [5,6], and immunohistochemical detection of Bcl2, p53 and cytokeratins, in human and canine tumors and corresponding adjacent tissues, have been similar [8].

In dogs, standard treatment for mammary gland cancer is surgical excision however, chemotherapy recommendation, as well as in women, is based on some prognostic factors. Furthermore, clinical information available in veterinary medicine suggests that drugs that are effective in human breast cancer, such as doxorubicin, cyclophosphamide, 5-fluorouracil and taxanes, may play a role in the treatment of malignant mammary gland tumors in dogs [2,9-12].

In women, neoadjuvant chemotherapy for breast cancer is associated with the same survival benefit as adjuvant chemotherapy and offers the advantage of an increased likelihood of breast conservation. Many drug regimens have been used for a varied number of cycles and two of the most used drugs, doxorubicin and cyclophosphamide, when given before surgery are associated with 49–85% response rates [13-15].

Another potential benefit of neoadjuvant chemotherapy may be the opportunity of *in vivo *assessment of tumor response and the possibility of determination of potential predictive factors, which may influence clinical decision making in the future. However, this potential has yet to be fulfilled, and although predictive factors might help selection of the appropriate treatment for each individual patient, to date, there is no single marker with a predictive value for a patient's response to chemotherapy [16].

We have previously conducted a study to identify predictive markers of response to neoadjuvant chemotherapy based on doxorubicin. Forty-four breast cancer patients submitted to neoadjuvant chemotherapy (doxorubicin and cyclophosphamide, AC, for four cycles, each 21 days) had tumor samples collected before treatment. Response was evaluated by palpation of the breast tumor and axillary lymph nodes, before and after chemotherapy, and a reduction of at least 30% in tumor dimension was classified as a partial response, according to RECIST criteria [17]. Following these criteria, 35 and nine patients presented a responsive and a resistant disease, respectively. Tumor gene expression was evaluated by cDNA microarrays and a differential profile between responsive and non-responsive patients, was determined. In addition, an extensive search was done in order to select trios of genes, whose expression could separate the responsive versus non-responsive tumors. One such trio genes was PRSS11 (Protease, Serine, 11), MTSS1 (Metastasis Suppressor 1), and CLPTM1 (Cleft Lip- and Palate-Associated Transmembrane Protein 1), which could correctly classify 95% of the samples, and another one, was PRSS11, MTSS1, and SMYD2 (Set and Mynd Domain-Containing Protein 2) [18].

Our present aim was to evaluate whether expression of these trios of genes could also predict drug response in another animal species. However, neoadjuvant chemotherapy is not routinely administered to dogs, as mammary gland conservation is of limited value. An option would be to analyze tumor response to chemotherapy *in vitro*.

Increasing evidence indicates that tumor cell behavior depends upon dynamic interactions between epithelial tumor cells and their microenvironment, including stromal cells and extracellular matrix. In addition, breast cancer tissue maintained in short term culture was previously shown as a potential model to study the activity of drugs (i.e. paclitaxel) and hormones (i.e. estrogen and calcitriol) [19-22]. Hence, we have also examined whether response to chemotherapy could be evaluated in mammary carcinoma from dogs when cultured as tissue slices.

Our data indicate that expression of these two trios of genes is not associated with canine mammary carcinoma response to doxorubicin, however, tumor slices culture may be an interesting model to evaluate drug response *in vitro*.

## Methods

Tumor samples were obtained from 38 dogs undergoing mastectomy at the "Hospital da Faculdade de Medicina Veterinária da Universidade Metodista de São Paulo (UMESP)", São Bernardo do Campo, SP, Brazil, from March 2005 to January 2006. This study was approved by the Institutional Ethics Committee and animal owners signed the informed consent. Median age of patients was 10.4 y and 55% and 18.4% of them were mixed and poodle breeds, respectively. Eight patients were previously spayed.

Patients were evaluated by clinical history and physical examination including mammary tumor measurement and inguinal and axillary nodes palpation, performed by two veterinarians. Regional lymph nodes were dissected during surgery and submitted to histological examination. Thoracic radiographs (ventrodorsal, right-to-left and left-to-right lateral projections) were performed to detect pulmonary metastasis. Patients were classified in clinical stage III (39.4%), II (28.9%), I (18.4%) and IV (13.1%) (pulmonary metastasis only) [23].

After histological examination of the surgical specimens by a veterinary pathologist, only samples of infiltrating carcinoma were selected for RT-PCR analysis. Carcinomas were classified as complex (WHO class 1.2) or simple (WHO class 1.3), including tubulopapillary (tubular, papillary, or papillary-cystic types), solid and anaplastic carcinomas [24]. The most frequently histological type observed was tubullopapillary (tubular and cystic-papillary, 34.2% and 28,9%, respectively) (Table 1). No anaplastic carcinomas were detected. Tumors were mainly of low histological grade.

**Table 1:** Characteristics of patients.

**Patient**	**Age (y)**	**Breed**	**Previously spayed**	**T**	**N**	**M**	**Clinical stage**	**Tumor type**	**Histological Grade**
1	12	Mixed breed	Yes	3	(-)	0	III	TPC (PC)	I
2	8	Doberman Pinscher	No	3	(-)	1	IV	TPC (TC)	I
3	12	German Shepherd	No	3	(-)	0	III	TPC (TC)	III
4	13	Belgian Shepherd	No	3	(-)	0	III	CC	II
5	13	Mixed breed	No	1	(-)	0	I	TPC (TC)	I
6	8	Napolitan mastiff	No	1	(-)	0	I	TPC (TC)	I
7	12	Poodle	No	2	(-)	0	II	TPC (CPC)	I
8	10	Mixed breed	No	3	(-)	0	III	TPC (CPC)	II
9	11	Mixed breed	No	2	(-)	0	II	TPC (CPC)	I
10	7	Akita	No	3	(-)	x	III	TPC (CPC)	I
11	11	Akita	No	3	(-)	0	III	TPC (TC)	II
12	9	Mixed breed	No	2	(-)	0	II	TPC (TC)	II
13	15	Mixed breed	No	2	(-)	0	II	SC	II
14	13	Mixed breed	No	1	(-)	0	I	TPC	II
15	6	Mixed breed	No	3	(-)	0	III	SC	ND
16	12	Dachshund	No	3	(-)	0	III	SC	ND
17	10	Poodle	Yes	1	(-)	0	I	TPC (TC)	ND
18	12	Mixed breed	No	3	(+)	1	IV	TPC (TC)	I
19	15	Mixed breed	No	1	(-)	0	I	TPC (PC)	I
20	8	Poodle	No	2	(-)	0	II	TPC (CPC)	ND
21	11	Mixed breed	No	1	(-)	0	I	TPC (TC)	I
22	13	Mixed breed	No	3	(-)	ND	III	TPC (CPC)	I
23	13	Mixed breed	No	2	(-)	0	II	TPC (TC)	ND
24	11	Poodle	No	3	ND	0	III	TPC (CPC)	II
25	15	Mixed breed	Yes	3	(-)	0	III	TPC (CPC)	I
26	7	Mixed breed	No	3	(-)	0	III	TPC	ND
27	11	Mixed breed	Yes	3	(-)	1	IV	TPC	I
28	2	Mixed breed	Yes	2	(-)	0	II	SC	ND
29	13	Irish setter	No	3	(-)	0	III	TPC (TC)	I
30	14	Mixed breed	Yes	3	(-)	0	III	TPC	III
31	8	English Cocker Spaniel	No	3	(-)	0	III	TPC (CPC)	ND
32	8	Poodle	No	2	(-)	0	II	TPC	I
33	8	Poodle	No	2	(-)	0	II	TPC (CPC)	I
34	10	Poodle	Yes	2	(-)	1	IV	TPC	I
35	9	Rottweiler	Yes	2	(-)	0	II	TPC (TC)	I
36	7	Mixed breed	No	2	(-)	0	II	TPC (CPC)	ND
37	7	Akita	No	2	(+)	1	IV	TPC	III
38	13	Mixed breed	No	1	(-)	0	I	TPC (TC)	II

Clinical stage classification, according to Owen [23]. Tumor types: complex carcinoma (CC); tubulopapillary carcinoma (TPC), subdivided in tubular carcinoma (TC), papillary carcinoma (PC) and cystic-papillary carcinoma (CPC); and solid carcinoma (SC), according to Misdorp *et al*., [24]. Histological grade, according to Elston & Ellis [33]. ND: not determined; (-): absent; (+): present.

Fragments of approximately 10 mm wide × 20 mm long, from small as well as from bulky tumors, were collected just after surgery by tumor incision and placed into culture medium (DMEM with antibiotics and fungicide) for transportation. Fragments were further cut in consecutive 0.3–0.4 mm-thick slices, using the Krumdieck tissue slicer (Alabama Research and Development Corporation, Birmingham, AL, USA) [20]. Four to six tumor slices were then cultured into two Petri dishes (90 × 15 mm), one containing just culture medium (10 mL RPMI, supplemented with 10% bovine fetal serum and 100 U/mL ampicillin, 100 mg/mL streptomycin) and the other one, also containing doxorubicin (1 μM) at 37°C in a humidified atmosphere of 95% air, 5% CO_2_, for 24 h. After the treatment period, one slice of tissue was fixed in buffered formalin for histological analysis and cell counting and the other slices were cryopreserved in liquid nitrogen for molecular analysis. Infiltrative cancer was represented on all samples analyzed as verified by histological analysis.

**Figure 1:**
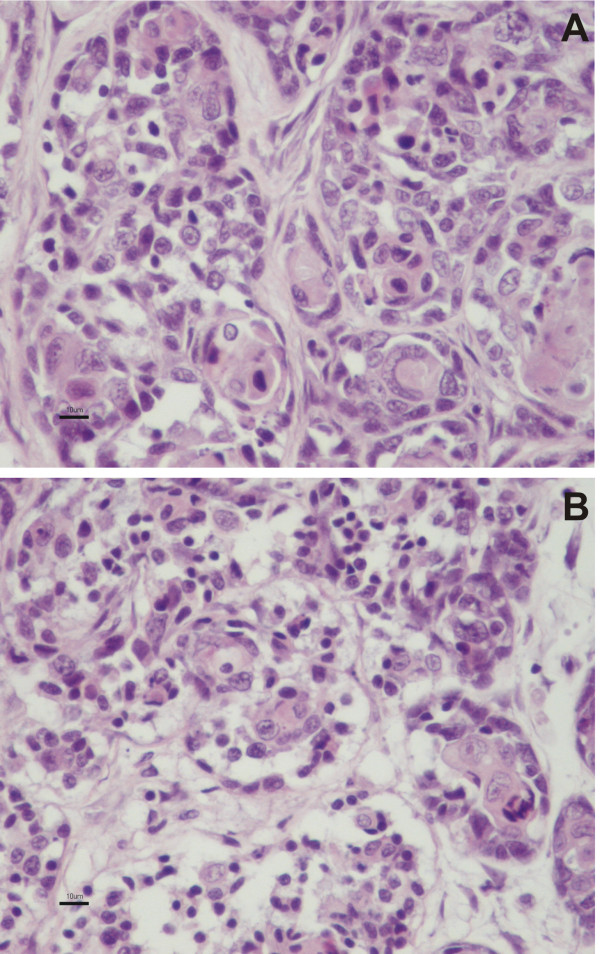
**Specimens maintained in culture medium and unexposed (A) or exposed (B) to doxorubicin for 24 h.** Mammary gland tissue is well preserved upon culture. Bar = 10 μm.

Response was evaluated by cell counting in paraffin embedded and hematoxilin-eosin stained slides of untreated (control) and corresponding doxorubicin treated tissue specimens (Figure 1). For this examination ten circles of 2 mm diameter were randomly drawn over the glass slides and encircled tumor cells were counted, using a Nikon Eclipse E-600 microscope (Nikon Instruments Inc, Melville, NY, USA).

At first, 16 samples had their cell number counted by two observers (RS and STH), to establish the inter-observer variation. Both observers counted all tumor cells inside the ten circles, and a mean value was calculated, which was considered 100% (example, RS: 750 cells and STH: 830 cells, mean 790 cells = 100%). The difference between cells counted by observers and the mean, was determined as percentage of variation (ex: difference observers and mean: 40 cells = 5.0% variation). A positive correlation was observed between the two observers (r = 0.797, *P *< 0.001, Spearman correlation) and mean, median and 75 percentage variations in cell counting between them were 13.8%, 11.75% and 21.7%, respectively.

These calculations were used to establish a cut-off value to define response to chemotherapy. We assumed that a reduction in cell number between doxorubicin treated and control samples superior to the 75 percentage variation in cell counting by different observers (21.7%) would be significant. Hence, we have arbitrarily adopted a reduction of 21.7%, as the cut-off value to define response.

All 38 samples maintained in cell culture and untreated or treated with doxorubicin had their tumor cells counted. The difference in tumor cell number between samples was expressed as percentage of variation [(cell number of treated sample – cell number of untreated sample) × 100/cell number of untreated sample]. Responsive samples were those presenting a reduction in the number of cells equal or higher than 21.7%, between treated and untreated samples (Table 2).

**Table 2:** Tumor response to doxorubicin *in vitro *treatment.

**Patient**	**Cell number in control samples**	**Cell number in treated samples**	**Cell number variation (%)**	**Response**
1	2307	1543	- 33,12	R
2	335	297	- 11,34	NR
3	2611	2472	- 5,32	NR
4	2800	2575	- 8,04	NR
5	472	216	- 54,24	R
6	445	278	- 37,53	R
7	354	304	- 14,12	NR
8	1339	1412	+ 5,45	NR
9	1581	1337	- 15,43	NR
10	1405	625	- 55,52	R
11	644	656	+ 1,86	NR
12	699	700	+ 0,14	NR
13	5414	5086	- 6,06	NR
14	2816	2602	- 7,60	NR
15	1268	1095	- 13,64	NR
16	1851	1644	- 11,18	NR
17	11189	9691	- 13,39	NR
18	4964	3713	- 25,20	R
19	1047	1031	- 1,53	NR
20	1869	1656	- 11,40	NR
21	1629	1199	- 26,40	R
22	1675	1234	- 26,33	R
23	1879	1722	- 8,36	NR
24	2155	2103	- 2,41	NR
25	668	576	- 13,77	NR
26	4849	4262	- 12,11	NR
27	3329	3266	- 1,89	NR
28	4376	3396	- 22,39	R
29	3559	2863	- 19,56	NR
30	2716	2932	+ 7,95	NR
31	4605	3569	- 22,50	R
32	5025	4242	- 15,58	NR
33	4239	4147	- 2,17	NR
34	4334	4146	- 4,34	NR
35	3691	4131	+ 11,92	NR
36	5841	4659	- 20,24	NR
37	2201	2256	+ 2,50	NR
38	5578	4533	- 18,73	NR

Cell number was counted in control (untreated) and doxorubicin treated samples. The signal (-) stands for the percentage cell reduction and (+) for the percentage cell increase, in treated as compared to control samples. R: responsive (reduction in cell number ≥ 21.7%); NR: non-responsive (reduction < 21.7%).

### Total RNA from frozen specimens

Gene expression was determined in cultured slices not exposed to doxorubicin, in accordance to our previous work, in which gene expression was determined in tumor biopsies, collected before the neoadjuvant treatment [18].

Tissue specimens were pulverized (Bio-Pulverizer™ BioSpec Products Inc., OK, USA) under liquid nitrogen and total RNA was isolated using Trizol reagent (Invitrogen Corporation, Carlsbad, CA, USA), according to the manufacturer's protocol. All RNA samples were treated with DNaseI for 30 min at 37°C to eliminate genomic DNA contamination. RNA quality and integrity was verified by the Absorbance A_260/280_, which varied between 1.78 and 2.0, and through observation of 28S/18S rRNA on agarose gel (1%) electrophoresis in denaturant conditions (ratio > 1.5).

### Real-time quantitative reverse transcription-polymerase chain reaction

Two micrograms of total RNA was reverse-transcribed using oligo(dT) primer and Superscript II (Invitrogen). Real-time (RT)-PCR was performed using SYBR-green I (Sigma, St. Louis, MO, USA) in a Rotor-gene system (Corbett Research, Mortlake, Australia).

PCR primer sets for SYBR-green I RT-PCR were designed based on the full-length sequences from exons, separated by introns, preferentially located in the coding region, closer to the 3' end of the gene (Table 3) using the software Primer3 . All sequences were specific for *Canis lupus familiaris*.

**Table 3:** Primer sequences of genes of interest. Sequences were obtained from *Canis lupus familiaris*.

*Gene*	*GenBank Accession number*		*primer sequence*	*Product size*
PRSS11	XM_535044	Sense	TGCTTTCGGAGCGTATATC	159 bp
		Anti-sense	CCATGTTCAGGGTGTTCTCC	
MTSS1	XM_539158	Sense	GACTCCCTTCAGTGCTCCAG	189 bp
		Anti-sense	CCGGTAAGACTGGCTGATGT	
CLPTM1	XM_541570	Sense	TGAGGGCCTTGTAAGTGAGC	151 bp
		Anti-sense	CACAAGGGCTGGTACTCCTG	
SMYD2	XM_537149	Sense	GCTTGTACATGCAGGACTGG	202 bp
		Anti-sense	CCGTGAGCCACTTCCATTAT	
GAPDH	NM_001003142	Sense	GGGTCATCATCTCTGCTCCT	150 bp
		Anti-sense	AGTGGTCATGGATGACTTTGG	

Amplification reactions were carried out using 2 μL cDNA diluted 1:10 (final volume of 20 μL), 1.25 units Platinum Taq Polymerase (Invitrogen), 1× polymerase buffer (Invitrogen), 2.0 mM MgCl2, 200 μM each dNTP, 0.2 μM each primer, 5% DMSO, 0.5 μL BSA 10 mg/mL (Promega Corp., Madison, WI, USA), and 0.1 μL SYBR^® ^Green. Amplification conditions consisted of denaturation at 95°C for 15 s followed by 40 cycles for annealing at 60°C for 60 s, and extension at 72°C for 60 s.

Relative expression of the genes of interest was calculated based on the expression of the endogenous housekeeping gene GAPDH. A pool of six samples from canine mammary tissue, collected from a mammary gland far away from the primary tumor site and not affected by any kind of tumor, was considered as a reference sample in all determinations. Reactions were performed in duplicate and CT variation between them was < 1.5. Analysis was performed as recommended by Pfaffl [25] using the efficiency value of the reaction and the CT value.

Relative expression results were then transformed to their natural logarithm values. Tumor specimens were then spatially disposed according to the expression of trios of genes. Fisher linear discrimination test was used to generate a separation plane between responsive and non-responsive samples.

## Results

Based on the previous established response criterion, a reduction in the cell number ≥ 21.7% upon doxorubicin treatment, nine samples were considered responsive to doxorubicin and 29 non-responsive (Table 2). In addition, considering the 38 samples treated and untreated, a mean reduction of 13.6% in the cell number (*P *< 0.001, Mann-Whitney test) was observed upon treatment.

**Figure 2:**
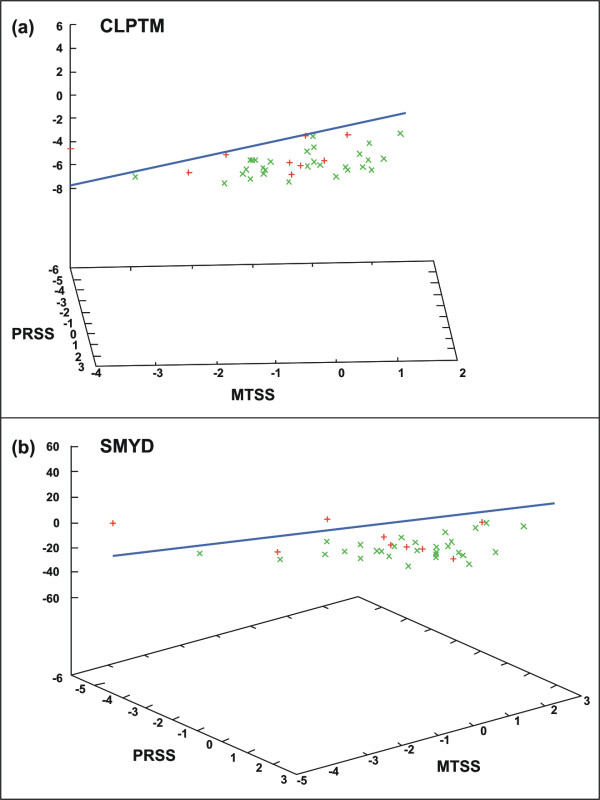
**Three-dimensional distribution of tumor samples according to expression of three genes: (a) PRSS11, MTSS1, CLPTM1 and (b) PRSS11, MTSS1, SMYD2. **Tumor response was defined as a reduction in cell number ≥ 21,7%. Each tumor is represented by a signal: green cross (non-responsive tumors, N = 29), red cross (responsive tumors, N = 9). Relative gene expression is shown on the axis as its natural logarithm value. Fisher linear discrimination test was used to generate a separation plane represented in blue.

Expression of PRSS11, MTSS1, CLPTM1 and SMYD2 was determined in tumor samples. Distribution of samples according to the expression of two trios of genes PRSS11, MTSS1, CLPTM1 and PRSS11, MTSS1, SMYD2, was then verified, in an attempt to separate responsive from non-responsive tumors. However, we could not verify a clear separation of tumors according to response to treatment (Figure 2).

As we adopted a very strict parameter to consider response to treatment (cell reduction ≥ 21.7%), we have also determined gene expression, considering the median percentage variation of cell counting between observers (11.7%) as the cut-off value of drug response. Using this parameter, 18 samples would be considered responsive and 20 non-responsive. However, three dimension distribution of samples based on the expression of the same two trios of genes could not separate tumors, according to response to doxorubicin (data not shown).

## Discussion

Tumor slices cultured *in vitro *may be an interesting model to evaluate drug response as it preserves some of the *in vivo *characteristics, as the epithelial mesenchymal relationship. An important issue is to guarantee a proper diffusion of oxygen and nutrients to the entire slice, as passive diffusion occurs through only 200 μm. In our study, tumor thickness varied between 300–400 μm and each tumor slice was placed on wells filled with culture medium, allowing them to float; conditions which, were previously shown to be appropriate to organ culture [19-22].

Slices were exposed to doxorubicin at a concentration of 1 μM, which equals the therapeutic dose in dogs. In addition, a similar concentration (0.84 μM) was shown to be the 50% inhibitory concentration in cell culture of mammary gland tumors, obtained from dogs [12]. Hence, an appropriate drug concentration for dogs was used.

In the present study, nine of 38 samples (23.6%) were classified as responsive to treatment. This response rate was inferior to that observed in women with breast cancer, submitted to neoadjuvant chemotherapy consisting of 4 cycles of anthracyclines, whose objective clinical response may vary between 49 and 85% [13,15,26]. Partial clinical response is defined as a tumor reduction ≥ 30%, evaluated by tumor dimension, according to RECIST criteria [17]. However, the high clinical response rate (49–85%) was observed after four cycles of neoadjuvant treatment, whether a low rate (23.6%), as we have observed, might reflect a single 24 h exposure.

Another aspect to take into consideration is the tumor histological grade. In women, increased clinical response rates were associated with high histological grade [27,28]. The histological grade seems to be of prognostic value in canine mammary carcinoma patients as in human patients [29]. However, in the present series, 47% of the tumors were low grade, which may have contributed to a low response rate.

Clinical response measured as a reduction in tumor dimension reflects a decrease in tumor cell number. We observed a mean reduction of 13.6% on the cell number and, in accordance to our data, Ciftci *et al*. [30] observed a reduction between 12–16% while analyzing human breast normal epithelial (MCF10) and cancer lineages (MCF7, MDA) using the same concentration of doxorubicin. Thus, we believe that the results of our study reflect an initial response after a short period treatment.

In the present series, the expression of trios of genes MTSS1, PRSS11, CLPTM1 and MTSS1, PRSS11, SMYD2, could not cluster canine samples according to response to doxorubicin. Recent studies indicate that tumors with diverse prognosis present a characteristic gene expression. According to this hypothesis, the primary tumor expression profile may identify patients with an indolent disease from those with an aggressive disease [31,32]. Our previous study in breast cancer patients treated with neoadjuvant AC included mainly women with advanced disease. Comparing tumor grades in different species is not straight forward as clinical stage criteria differ between animal species. However a certain level of comparison is possible. In the present series, 39% of the dogs presented in clinical stage III, 5% had lymph node metastasis and 13% presented pulmonary metastasis, as compared to 80%, 75% and none, respectively, considering the women patients [18]. Hence, as clinical stage is a powerful prognostic factor and as tumor transcriptome varies among tumors with differential prognosis [31,32], it could be inferred that early and advanced stage tumors present a differential gene expression profile associated with doxorubicin response. Furthermore, in our current work, invasive tubular adenocarcinoma and invasive solid carcinoma, which are associated with a poor prognosis [1,33] represented 43% of the specimens, and these histological types might have been an adequate model to study aggressive tumors in dogs. Finally, inter-species genetic heterogeneity is another factor that could have contributed to determine a diverse gene expression associated with response to chemotherapy.

It is important to emphasize that an *ex-vivo *model of tissue slice culture, where epithelial-mesenchymal interactions are maintained, may add information to a model where isolated cells are cultured. In addition, an *ex-vivo *model allows a closer evaluation of cell heterogeneity associated with each individual tumor. However, although this model may be useful to study some aspects underlying chemotherapy response, conclusive data on predictive factors deserves further validation through clinical studies where patients receive chemotherapy.

## Conclusion

Our data suggest that short term culture of mammary tumor slices seems to be an interesting model to evaluate doxorubicin activity. However, parallel comparisons between *in vitro *and *in vivo *drug responses to establish their exact correlation are needed. Moreover, our results on the expression of a few genes emphasize the need to obtain a more detailed gene expression profile, associated with chemotherapy response in canine tumors.

## Authors' contributions

RAS participated in the design of the study, sample collection, tissue slice culture, PCR assays, and helped to draft the manuscript. STH participated in sample collection, tissue slice culture, and cell counting. MLHK participated in the design of the study, tissue slice culture. PCR assays and revised the manuscript for important intellectual content. HB participated in the design of the study and performed statistical analysis and data interpretation. MMB participated in the design of the study and revised the manuscript for important intellectual content. DFCP performed statistical analysis. MAAKF participated in the design of the study, data interpretation and helped to draft the manuscript.
